# Intratumoral microbiome: implications for immune modulation and innovative therapeutic strategies in cancer

**DOI:** 10.1186/s12929-025-01117-x

**Published:** 2025-02-19

**Authors:** Na Wang, Si Wu, Lanxiang Huang, Yue Hu, Xin He, Jourong He, Ben Hu, Yaqi Xu, Yuan Rong, Chunhui Yuan, Xiantao Zeng, Fubing Wang

**Affiliations:** 1 Department of Laboratory Medicine, Zhongnan Hospital of Wuhan University, Wuhan, 430071 China; 2 Center for Single-Cell Omics and Tumor Liquid Biopsy, Zhongnan Hospital of Wuhan University, Wuhan, 430071 China; 3 Center for Tumor Precision Diagnosis, Zhongnan Hospital of Wuhan University, Wuhan, 430071 China; 4 Department of Laboratory Medicine, Wuhan Children’s Hospital (Wuhan Maternal and Child Healthcare Hospital), Tongji Medical College, Huazhong University of Science & Technology, Wuhan, 430016 China; 5 Center for Evidence-Based and Translational Medicine, Zhongnan Hospital of Wuhan University, Wuhan, 430071 China; 6 Department of Urology, Zhongnan Hospital of Wuhan University, Wuhan, 430071 China; 7 Wuhan Research Center for Infectious Diseases and Cancer, Chinese Academy of Medical Sciences, Wuhan, 430071 China

**Keywords:** Intratumoral microbiome, Omics technology, Immunomodulation, Microbiome-immune crosstalk, Immunotherapy, Therapeutic interventions

## Abstract

Recent advancements have revealed the presence of a microbiome within tumor tissues, underscoring the crucial role of the tumor microbiome in the tumor ecosystem. This review delves into the characteristics of the intratumoral microbiome, underscoring its dual role in modulating immune responses and its potential to both suppress and promote tumor growth. We examine state-of-the-art techniques for detecting and analyzing intratumoral bacteria, with a particular focus on their interactions with the immune system and the resulting implications for cancer prognosis and treatment. By elucidating the intricate crosstalk between the intratumoral microbiome and the host immune system, we aim to uncover novel therapeutic strategies that enhance the efficacy of cancer treatments. Additionally, this review addresses the existing challenges and future prospects within this burgeoning field, advocating for the integration of microbiome research into comprehensive cancer therapy frameworks.

**Graphical Abstract:**

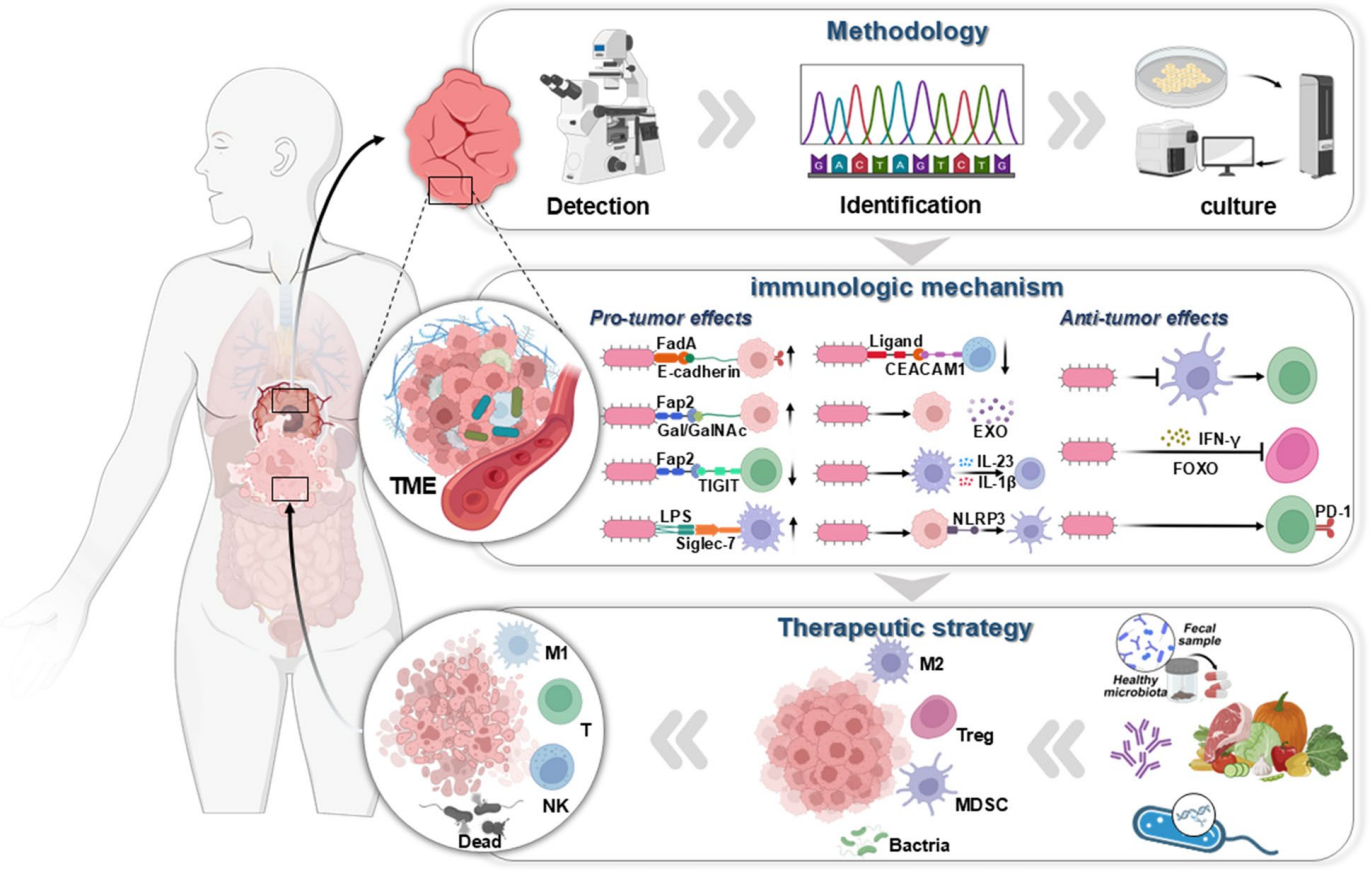

## Introduction

The characteristics of survival, proliferation, metastasis, and therapeutic response of tumor cells are not only driven by their intrinsic nature but are also greatly influenced by their interactions with the microenvironment components. One important component of the microenvironment is the recently recognized tumor microbiome, which is the latest breakthrough in understanding the tumor ecosystem. The presence of microbiomes has been confirmed in tumor masses that were previously thought to be sterile [1]. Microbiomes can interact with host cells and affect tumor development through a variety of mechanisms [2, 3]. Notably, tumor microbiota can mediate communication between the immune system and host cells [4]. A coevolutionary relationship exists between the tumor microbiome and the innate or adaptive immune system, which can affect tumor progression through direct and indirect interactions between tumor and immune cells, such as the induction of metabolic inflammation [5]. Furthermore, host-microbial characteristics can predict tumorigenesis, modulate the efficacy and toxicity of tumor immunotherapy, and predict the prognosis of patients with tumors [6–8]. In this review, we introduce the characteristics of the intratumoral microbiome, summarize existing and potentially applicable research techniques in the field of the tumor microbiome, emphasize the immune regulatory role of the intratumoral microbiome, and discuss approaches to enhance the efficacy of cancer therapy through the modulation of the intratumoral microbiome. Finally, we discuss the challenges and prospects of studying the intratumoral microbiome.

## Detection, identification, and culture of the intratumoral microbiome

**Fig. 1:**
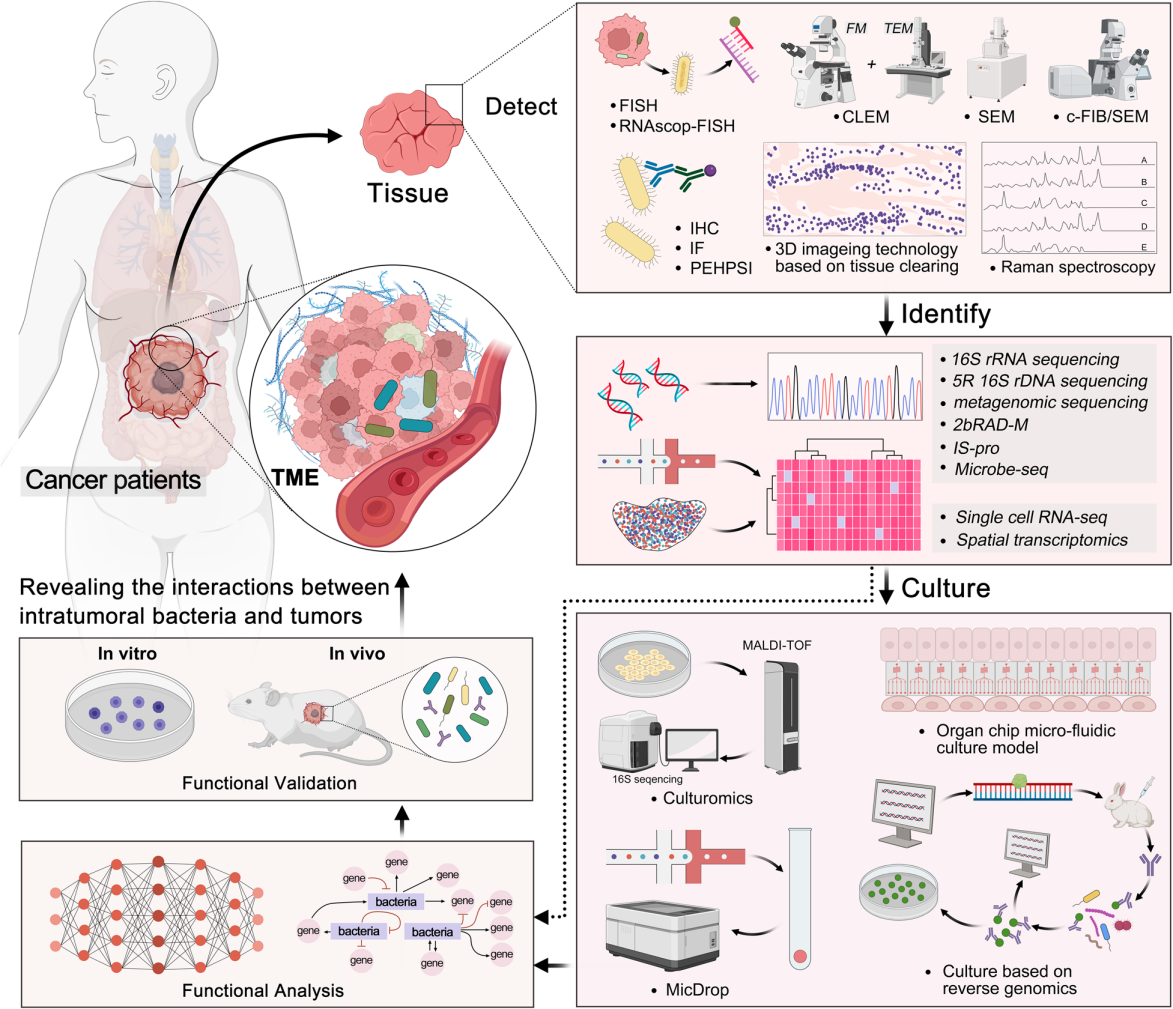
Promising technologies for the detection, identification, and culture of intratumoral microbiome for revealing the interactions between intratumoral bacteria and tumors

Since the discovery of microbiota within tumors, there has been a rapid influx of researchers aiming to elucidate their roles in tumors. Although some progress has been made, with the tumor microbiome displaying considerable potential as both a diagnostic biomarker and a therapeutic target in cancer, the limited biomass of these microbes has impeded the advancement of related research. To enhance the feasibility of utilizing intratumoral microbiomes in tumor diagnosis and treatment, it is imperative to precisely detect and identify low-abundance microbial populations within tumors, followed by their cultivation for the analysis and verification of their biological functions, thereby revealing microbiota-host interactions in the tumor microenvironment (Fig. 1). In recent years, alongside conventional methods for microbial detection, identification, and culture, novel approaches have emerged to meet the research demands concerning tumor microbiomes. The subsequent sections offer a comprehensive overview of established and recently developed methods and their applications for the detection, identification, and cultivation of tumor microbiomes (Table 1).

**Table 1:** Methods for the detection, identification, and culture of the tumoral microbiome

Classification	Strategy	Methods	Advantages	Disadvantage	Application	References
Detection	In situ hybridization	FISH	High sensitivity and specificity	Low signal strength	Locating microbes within tumor tissues	[11, 17]
		RNA scope-FISH	Single-cell resolution	Expensive, False positive	Visual detection of *Fusobacterium nucleatum* in colorectal cancer	[13, 14]
	Specific binding between antigens and antibodies	IHC	Convenient and rapid operation	Color superposition	Validate the presence of bacteria in human tumors	[1, 56]
		IF	Convenient and rapid operation	Fluorophore spectral overlap	Bacterial colonization sites in tumor tissues were labeled	[15, 54]
		PEHPSI	Multiplex, Rapid, Semi-quantitative	Expensive, Complex probe design	Characterize bacteria, immune and breast cancer cell subtypes within the same solid tumor tissue sections	[16]
	Microscope	TEM	High resolution, Imaging internal structures, Clear images	Expensive, Complex operation	To visualize the morphology of bacteria encapsulated within lysosomes and the outline of bacteria being digested by lysosomes in the tumor tissues of intrahepatic cholangiocarcinoma (ICC)	[11]
		SEM	High resolution and magnification, High depth of field	Inability to observe internal details and fine structures of complex samples	Observe *streptococcus* in the tumor tissues of ESCC	[15]
		CLEM	High specificity and resolution, Multicolor fluorescence imaging	Expensive, Complex operation	To confirm that bacteria can enter melanoma cells	[17]
		C-FIB/SEM	High resolution, 3D analysis	Expensive, low throughput	Investigate host-microbe interactions at the 3D ultrastructural level	[18]
	3D imaging technology based on tissue clearing	MiPACT-HCR	High sensitivity, Complex sample analysis	Complex sample processing, non-specific binding	Visualize bacteria in transparent sputum samples	[21]
		TiDaL	Single-bacteria resolution	Inaccurate bacterial localization	To detect metabolically active bacteria and enable 3D imaging of the microbiota within small intestinal crypts	[57]
		3D quantitative in situ imaging	3D, situ imaging	Low accuracy and sensitivity	Visualize of bacterial LPS fluorescence signals in human glioma samples	[23]
	Vibrational spectrum	Raman spectroscopy	High specificity and spatial resolution	High costs and background interference	Detect the distribution of *Staphylococcus aureus* within endothelial cells	[26]
		CAST-R-HP	Single-cell resolution	High technical requirements, Single test sample	Rapid detection of *H. Pylori* in clinical biopsy samples	[29]
Identification	Genome sequencing	16S rRNA gene sequencing	High throughput, Low cost, Low detection limit	The resolution cannot reach the species level	Widely used to identify tumor microbiomes	[11, 14, 17, 58]
		5R 16S rDNA sequencing	Species-level resolution	High costs and technically demanding	Identify unique microbiomes with high coverage and resolution for different types of cancer, for example, breast cancer is characterized by a rich and diverse microbial profile	[1]
		Metagenomic sequencing	Capable of identifying microorganisms at the species or strain level	High costs	To identify the specific microbiome in cancer tissues	[34, 35]
		2bRAD-M	Capable of handling samples with low biomass, high host contamination, and severe degradation	Limited by reference genome availability and technical needs	Revealing differences in microbial distribution between ovarian cancer and benign ovarian tissues	[37]
		IS-pro	Fast and can be identified at the species level	Expensive, Proprietary technology	Rapidly identified most of the important lung pathogens, such as *Burkholderia* and *Pseudomonas*	[38]
		Microbe-seq	High-throughput, Single-cell resolution	Expensive, Proprietary technology	Analyze single bacterial cells from a microbiota	[40]
	Transcriptome sequencing	PETRI-seq	High specificity and sensitivity	Complex sample processing, Cross-contamination	Capture single cell transcriptomes of Gram-negative and Gram-positive bacteria	[42]
		MicroSPLiT	High specificity, multiplex detection	High cost, Limited throughput	Reveal gene expression states in bacteria	[43]
		smRandom-seq	High specificity, Minor doublet rate, Sensitive gene detection	Expensive, Proprietary technology	Capture the transcriptome changes of thousands of individual *E. coli* and discover a few antibiotic-resistant subpopulations	[41]
		BacDrop	High throughout, Utility	Expensive, Limited in complex bacterial communities	Reveal transcriptionally distinct subpopulations associated with different phenotypic outcomes	[44]
		INVADEseq	Single-cell resolution, bacteria–host interactions analysis	Expensive, Proprietary technology	Identify cell-associated bacteria and the host cells with which they interact, as well as relate their functions	[14]
		scDual-Seq	Bacteria–host interactions analysis	Limitation of a high MOI	Investigate the process of infection of individual mouse macrophages with the intracellular pathogen *Salmonella typhimurium*	[45]
		SAHMI	Bacteria–host interactions analysis	Not suitable for specific, rare, or hard-to-find taxa	Reveal the characteristics of tumor-microbiome interactions in pancreatic cancer	[46]
Culture	/	Culturomics	High-throughput, Repeatability	Time-consuming, Labor-intensive, and technically demanding	To culture *Gemella sanguinis* and *Streptococcus intermedius* from the tumor tissues of non-small-cell lung cancer patients, while isolating *Prevotella* from healthy oral cavities to establish a sample bank of lung and oral microbiomes for lung cancer patients	[1, 51]
	/	MicDrop	High-throughput	False-positive due to multicellular encapsulations	Isolation and cultivation of individual bacteria from the human microbiota and assessment of the growth and function of microbiota components	[53]
	/	Organ chip microfluidic culture model	Bacteria–host interactions analysis	Unable to maintain long-term cultures	Create a preclinical model to understand the interactions between the vaginal microbiome and host tissues	[54]
	/	Reverse genomics	applied to the culture of any species in any environment and can unlock new species	Limited by bacterial abundance	To isolate and cultivate three distinct species of *Saccharibacteria* from the oral cavity as well as previously uncultured *SR1* bacteria	[55]

## Detection methods

### Methods based on in situ hybridization

Fluorescence in situ hybridization (FISH) is a widely employed molecular cytogenetic technique for the detection, identification, and localization of bacterial 16S rRNA genes using highly complementary fluorescent DNA probes with high sensitivity and specificity [9, 10]. FISH plays a crucial role in the detection of intratumoral microbiota. Chai et al*.* successfully employed FISH to detect bacteria such as *Klebsiella pneumoniae* and *Paraburkholderia fungorum* in intrahepatic cholangiocarcinoma [11]. Nevertheless, the original FISH method often exhibits a low signal strength because of inadequate cell permeability and limited nucleic acid copy numbers [12]. Therefore, a novel FISH technique called RNAscope-FISH has been developed, which can amplify signals while suppressing background noise, enabling the visualization of individual molecules within a single cell. This technique has been successfully applied to confirm the heterogeneous spatial distribution of bacterial communities, such as *Fusobacterium nucleatum* (*F. nucleatum*), in colorectal cancer (CRC) [13, 14].

### Methods based on immunology

Immunohistochemistry (IHC) and immunofluorescence (IF) techniques rely on the principle of specific binding between antigens and antibodies. In IHC, labeled antibodies produce color through chemical reactions, whereas IF utilizes fluorescent materials to qualitatively and quantitatively detect antigens in tissues. Currently, both technologies are used to detect bacteria in tumor tissue sections. Wu et al*.* successfully labeled bacterial colonization sites in tumor and para-carcinoma tissues of esophageal squamous cell carcinoma using antibodies against bacterial lipopolysaccharides (LPS) and lipoteichoic acid (LTA) [15]. Additionally, researchers further optimized and improved immunology to develop a multiplex rapid semi-quantitative method, prokaryotic and eukaryotic cell hybrid probes for in situ imaging (PEHPSI), which can not only characterize bacteria but also detect immune and breast cancer cell subtypes. Using this technology, it was found that immune cells or breast cancer cell functional biomarkers in bacterial-rich areas of different breast cancer subtypes were upregulated or downregulated to varying degrees [16], which provides an alternative method to analyze the crosstalk among cancer cells, immune cells, and intratumoral bacteria.

### Methods based on microscope

Fluorescence microscopy can be used to determine the presence of bacteria by identifying fluorescently labeled tumor tissue sections; however, it does not provide visualization of the bacteria. In contrast, electron microscopy enables observation of bacterial morphology. Chai et al*.* employed transmission electron microscopy (TEM) to observe bacteria in the tumor tissues of intrahepatic cholangiocarcinoma and could visualize the morphology of bacteria encapsulated within the lysosome and the outline of the bacteria digested by the lysosome [11]. Scanning electron microscopy (SEM) is also applicable and is commonly employed to observe culturable bacteria in tumor tissues [15]; however, it has limitations in observing complex samples and internal fine structures. Correlative light and electron microscopy (CLEM) effectively combines the advantages of fluorescence microscopy and electron microscopy, while minimizing their limitations. This enables the acquisition of cellular functional and ultrastructural information with high specificity and resolution, thereby facilitating the localization of intratumoral bacteria. Kalaora et al*.* used CLEM to confirm that bacteria can enter melanoma cells [17]. This technique has emerged as one of the most effective options for observing bacteria in tumors. Furthermore, the recently emerged correlative focused ion beam/scanning electron microscopy (c-FIB/SEM) combines multidimensional fluorescence microscopy and volume electron microscopy to investigate host-microbe interactions at the 3D ultrastructural level [18]. This technique is expected to be demonstrated in future studies on the tumor microbiome.

### 3D imaging technology based on tissue clearing

Tissue-clearing techniques render the entire tissue transparent and minimize light scattering and absorption, thereby improving optical imaging and enabling the 3D visualization of tissues and organs [19, 20]. Currently, tissue-clearing techniques and bacterial labeling are extensively employed for in situ 3D imaging of microbiota. For example, microbial identification using the passive clarity technique with hybridization chain reaction (MiPACT-HCR) can visualize bacteria in transparent sputum samples [21]. A strategy based on tissue clearance and d-amino acid labeling (TiDaL), which detects metabolically active bacteria, enables 3D imaging of the microbiota within small intestinal crypts that were previously considered sterile [22]. To further investigate intratumoral bacteria, researchers have integrated tissue clearing, immunofluorescence labeling, optical sectioning microscopy, and image processing to develop a three-dimensional (3D) quantitative in situ intratumoral microbiome imaging strategy. This approach enables the visualization of bacterial LPS fluorescence signals in human glioma samples [23]. These continually optimized and enhanced imaging techniques have opened up new possibilities for imaging low-biomass intratumoral bacteria.

### Raman spectrum

Raman spectroscopy, utilizing a vibrational spectrum, enables noninvasive and label-free detection of bacteria by comparing the bacterial chemical composition or bacterium-secreted compounds [24]. This technique is commonly used to investigate microbial phenotypes and function [25]. By leveraging the high specificity and spatial resolution of Raman microspectroscopy, researchers have successfully detected the distribution of *Staphylococcus aureus* within endothelial cells [26] and determined the location of *Mycobacterium gordonae* in macrophages. The potential of Raman spectroscopy for investigating host niche dynamics has also been confirmed [27]. In recent years, microfluidic and flow cytometry-based single-cell Raman technologies have been developed to provide abundant intrinsic information about cells and reflect their genotype, phenotype, and physiological state [28]. The Clinical Antimicrobial Susceptibility Test Ramanometry for *H. pylori* (CAST-R-HP) technique, based on single-cell Raman spectroscopy, enables the rapid detection of *H. pylori* in clinical biopsy samples. Establishing a connection between the metabolic phenotype and the corresponding resistance genotype at a single-cell resolution facilitates the rapid diagnosis and treatment of *H. pylori* infection [29], which lays the foundation for its application in the field of tumor microbiome research.

## Identification methods

### Methods based on genome sequencing

Genome sequencing includes metagenomic, whole-exome, and targeted sequencing; among these, 16S rRNA gene sequencing and metagenomic sequencing are the most frequently employed techniques for identifying the tumor microbiome. The 16S rRNA is a component of the 30S subunit of bacterial and archaeal ribosomes and is highly conserved and specific. Hypervariable regions can reflect interspecific differences and identify bacterial species, among which hypervariable region 4 (V4) exhibits the highest specificity and is the optimal choice for bacterial diversity analyses [30, 31]. However, sequencing the 16S rRNA gene in a single variable region presents issues such as primer bias and limited coverage [32]. Consequently, multiple 16S rDNA sequencing approaches have been developed, such as 5R 16S rDNA sequencing, which enable the identification of the microbiome within breast tumors with high coverage and resolution [1]. 16S rRNA gene sequencing targets known bacteria and typically identifies them only at the genus level. In contrast, metagenomic sequencing can detect all DNA in tumor tissue samples, including all microorganisms and host DNA, enabling the identification of microorganisms at the species or strain level [10, 33–35]. However, this method incurs higher costs. Building upon this, researchers have developed a simplified metagenome technology named 2bRAD sequencing for microbiome (2bRAD-M), which enables cost-effective and efficient processing of samples with low microbial biomass, severe DNA degradation, and high host contamination. It provides qualitative and quantitative results at the species level for bacteria, archaea, and fungi [36]. This technique has been preliminarily applied in the field of tumor microbiome research to analyze the differences in microbial distribution between ovarian cancer and normal tissues [37]. Another PCR-based method, IS-pro, enables the rapid detection of bacterial DNA [38]. It can identify species, including those that have not been detected in culture, thereby potentially providing a more accurate representation of the actual microbial composition within a living body [39]. The analysis of different cell types and states at the single-cell level enables a more in-depth study of tumor microbiome heterogeneity. Microbe sequencing (microbe-seq), a single-cell microbial genome sequencing technology, has recently emerged. It integrates various drop microfluidic manipulation techniques and custom-developed bioinformatic analysis methods. Microbe-seq can be used to obtain genomic information from tens of thousands of single-cell microorganisms, without the need for culture. This technology provides an effective and practical approach for characterizing complex microbial communities at a single-cell resolution [40].

### Methods based on transcriptome sequencing

Since single-cell RNA-seq was first reported in 2009, this technique has rapidly developed and has found widespread application in the detection of cell diversity within complex eukaryotic tissues. However, the low mRNA content of bacteria and the lack of 3′-end poly(A) tails have posed significant challenges to single-cell transcriptomic detection of microorganisms [41]. Prokaryotic Expression-profiling by Tagging RNA In Situ and sequencing (PETRI-seq) [42] based on in situ combinatorial indexing and microbial split-pool ligation transcriptomics (MicroSPLiT) [43] based on split-pool barcoding solve this problem, enabling single-cell detection of both gram-positive and gram-negative bacteria. These techniques can also be used to analyze the heterogeneous transcriptional states of the microbiome. However, the throughput of these methods is limited, and the sequencing cost is high because of the presence of rRNA. Consequently, high-throughput and high-sensitivity microbial single-cell transcriptome sequencing technologies based on droplets, namely BacDrop and smRandom-seq, have been developed that can effectively eliminate rRNA and concentrate mRNA, achieve precise targeting of microbial-host interaction target subsets, and reduce sequencing costs by at least ten-fold [41, 44]. These advancements have significantly enhanced the prospects for clinical translation. Additionally, researchers have developed invasion-adhesion-directed expression sequencing (INVADEseq) and scDual-Seq, which enable the capture of transcriptome information from both hosts and bacteria. These techniques facilitate the identification of changes in the transcriptional pathways associated with cancer metastasis, DNA repair, and other processes [14, 45]. SAHMI is another recently developed single-cell analysis technique for studying host-microbiome interactions. It systematically recovers and filters contaminants from the host genome sequencing data, thereby obtaining the most accurate microbial signal. The developer further applied the technique to reveal the characteristics of tumor-microbiome interactions in pancreatic cancer at the single-cell level and to predict patient outcomes [46]. To gain a deeper understanding of the spatial heterogeneity within the tumor microbiome, spatially resolved co-detection of bacterial and host molecular markers and transcripts is required. Recently, newly developed spatial transcriptomic methods have enabled the acquisition of gene expression characteristics and spatial distribution data for both host and microbial species in situ. These methods have been successfully applied to oral squamous cell carcinoma, CRC, and lung cancer to assess host-microbiome interactions at spatial resolution [14, 47].

### Potential culture methods

Culture-based assays have the capacity to elucidate significant functional distinctions among individual microbial communities and help dissect differences in strain-level functional variation between individuals that drive health and disease outcomes. To gain a deeper understanding of the functions and mechanisms of intratumoral bacteria, it is crucial to obtain pure bacterial cultures. Culturomics, a high-throughput bacterial culture technique that employs various culture conditions, coupled with MALDI-TOF mass spectrometry or 16S rDNA sequencing for bacterial species identification [48], plays a pivotal role in elucidating the functions of specific host bacteria. Culturomics enables the discovery of potential new strains to a greater extent and enriches the existing culturable microbial resource bank [49]. Culturomics relies on changes in the culture conditions and media. In recent years, continuous workflow optimization has led to significant simplification of culture conditions while maintaining a high capture rate (98%) of the isolated strains [50]. Currently, researchers have employed culturomics to culture *Gemella sanguinis* and *Streptococcus intermedius* from the tumor tissues of non-small-cell lung cancer patients, while isolating *Prevotella* from healthy oral cavities to establish a sample bank of lung and oral microbiomes for lung cancer patients [51]. Although culturomics has been preliminarily applied to the isolation and culture of intratumoral bacteria, supporting evidence remains limited, and its reliability requires further investigation.

Given the harsh requirements of microbial culture environments for meeting research needs, numerous novel culture technologies have emerged in recent years to accommodate the growth of microorganisms in complex environments. Bioreactors can simulate the physiological conditions of the gastrointestinal tract in vitro, facilitating the proliferation and establishment of complex microbial communities. This provides a solution for cultivating microbes in vitro [52]. MicDrop, a culture platform that combines droplet microfluidics and high-throughput DNA sequencing, allows the isolation and cultivation of individual bacteria from the human microbiota. It also enables assessment of the growth and function of microbiota components, offering a promising method for culturing tumor microbiomes with low biomass [53]. In addition to the gut microbiome, the vaginal microbiome can be cultured by simulating the physiological environment. Mahajan et al*.* constructed an organ chip microfluidic culture model by recreating a vaginal epithelial environment. This model can be employed to evaluate optimal colonizing bacteria and host immune responses in the vagina [54]. This in vitro simulation method provides a reference for the cultivation of intratumoral bacteria by simulating the tumor microenvironment to create a suitable environment for the growth and reproduction of intratumoral bacteria, which may be inspired by organoid technology. Reverse genomics, another new technique that can be used to culture the tumor microbiome, utilizes a single-cell genome or metagenomic sequences to design antibodies targeting predicted cell surface proteins, enabling the cultivation of specific bacteria. Cross et al*.* successfully employed this technique to isolate and cultivate three distinct species of *Saccharibacteria* from the oral cavity as well as previously uncultured SR1 bacteria [55]. This technology can be applied to the culture of any species in any environment and can unlock new species, potentially providing a new technical solution for culturing intratumoral bacteria. Owing to the low biomass of the intratumoral microbiome, there is currently no reliable method to isolate low-biomass intratumoral bacteria and culture as-yet-uncultured microbes, which severely limits relevant research. More research is needed to explain the complex interactions between intratumoral bacteria and the tumor microenvironment, thereby providing essential theoretical groundwork for the development of culture techniques tailored specifically to the tumor microbiome.

## Intratumoral microbiome influence tumorigenesis and progression through immunomodulation

### Pro-tumor intratumoral microbiota: the malignant collaborators

Immune evasion is a hallmark of cancer [56]. In this context, the interplay between host microbes and the immune system represents a symbiotic physiological process. The anaerobic, nutrient-rich, and immunosuppressive milieu within tumors provides a setting that fosters symbiotic relationships among microbiomes, immune cells, and tumor cells. Intratumoral microbiomes can orchestrate the programming of the immune system into an inhibitory state, thereby bolstering growth and facilitating tumor development [57].

### F. nucleatum

When colonizing a tumor, bacteria with high site-specific colonization abilities can affect the responses of immune effector cells to malignant cells [58–60]. The first is the impact of intratumoral bacteria on tumor development, of which the most typical strain is *F. nucleatum*, which directly and/or indirectly suppresses the immune response to promote tumor growth. In CRC, intratumoral *F. nucleatum* promotes tumor development by inhibiting natural killer (NK) cell cytotoxicity and T cell activity [61–65], promoting the recruitment of tumor-associated macrophages (TAMs) [66–69], and inducing cytokine secretion by normal epithelial cells and tumor cells [70]. *Fusobacterium nucleatum* also can promotes colorectal cancer cell proliferation and tumor growth by activating TLR4 signaling to NF-κB and upregulating the expression of microRNA-21 [71]. In addition, Mandelboim, O. et al. found that *F. nucleatum* could bind and activate inhibitory receptors such as T cell immune receptor with immunoglobulin and ITIIM domain (TIGIT) and Carcinoembryonic antigen-related cell adhesion molecule 1 (CEACAM1) to inhibit natural killer (NK) cell cytotoxicity and T cell activity, thus enhancing colorectal cancer progression [64, 65]. Furthermore, FadA, a virulence protein of *F. nucleatum*, binds to E-cadherin to trigger a decrease in β-catenin phosphorylation and subsequent activation of β-catenin-regulated transcription, leading to increased oncogenic MYC expression, which upregulates the expression of immunosuppressive programmed cell death ligand 1 (PD-L1) and CD47 in cancer cells [72]. The derivatives of *F. nucleatum* also promote the progression of CRC through immunoregulation. Its outer membrane vesicles (OMVs) or LPS can induce pro-inflammatory responses in dendritic cells and tumor-associated responses in macrophages to promote CRC progression [73].

Beyond its established deleterious role in CRC, recent studies have expanded the scope of *F. nucleatum* to various other malignancies, providing new evidence for its pro-tumorigenic effects through immune modulation. In breast cancer, *F. nucleatum* leverages its lectin Fap2 to selectively colonize the TME, promoting tumor growth by inhibiting the accumulation of tumor-infiltrating T cells [74]. Similarly, in esophageal squamous cell carcinoma (ESCC), *F. nucleatum* infection and colonization attract an enrichment of Treg cells, which weakens antitumor immune responses and facilitates both its persistent colonization and the malignant progression of the tumor [75]. Moreover, in oral squamous cell carcinoma (OSCC), *F. nucleatum* residing in the tumor microenvironment (TME) triggers the GalNAc-autophagy-TBC1D5 signaling pathway, resulting in GLUT1 accumulation on the plasma membrane and extracellular lactate deposition. This cascade promotes tumor-associated macrophage (TAM) formation, further driving tumor progression [76]. As the oncogenic role of *F. nucleatum* within the intratumoral microbiome continues to emerge, it is increasingly recognized as a critical contributor to cancer progression. Targeting *F. nucleatum* may hold potential as a therapeutic strategy to enhance cancer treatment. However, before such interventions can be considered, a deeper understanding of the fundamental biology of *F. nucleatum* and its interactions with host cells and co-residing symbiotic microbiota is essential.

### *Bacteroides fragilis* (*B. fragilis*)

*Bacteroides fragilis* has been identified as a biomarker for poor prognosis in CRC, primarily exerting its effects through the secretion of toxin, particularly *Enterotoxigenic B. fragilis* (ETBF) strains. Reports indicate that CRC mouse models with intratumoral colonization of ETBF exhibit elevated levels of IL-17 and increased DNA damage within colonic epithelium, which accelerates tumor growth [77]. Furthermore, ETBF can induce Th17 cell differentiation via METTL14-dependent m^6^A methylation, downregulating tumor-derived exosomal miR-149-3p, thereby promoting CRC cell proliferation [78]. Additionally, *Bacteroides fragilis* tends to accumulate in CIMP (CpG island methylator phenotype)-positive tumor tissues and is significantly associated with CIMP characteristics such as MLH1 (mutL homolog 1) methylation [79]. Patients with high CIMP tumors often display features of immune suppression, including reduced immune infiltration, lower cytotoxicity, and diminished immune activation [80]. This immunosuppressive phenotype may potentially be mediated by intratumoral *B. fragilis*, although further research is required to elucidate this mechanism.

### *Helicobacter pylori* (*H. pylori*)

It is a well-established fact that *H. pylori* colonization in the stomach can induce chronic inflammation of the gastric mucosa and DNA damage, thereby leading to gastric cancer. This oncogenic potential is typically mediated by a variety of virulence factors, such as VacA (Vacuolating cytotoxin A), CagA (Cytotoxic- associated gene A), and OipA (Outer inflammatory protein A) [81]. VacA enhances the colonization capability of *H. pylori* in cancer cells and inhibits T-cell proliferation, disrupts B-cell antigen presentation, and alters macrophage signaling, thereby impairing the immune system's ability to eradicate *H. pylori* [82]. Intracellularly colonized *H. pylori* can utilize the virulence factor CagA to recruit PKC δ, promoting the phosphorylation of STAT3^S727^ within the nucleus, which upregulates IL-6 expression and accelerates the onset of gastric cancer [83]. CagA also activates NF-κB and induces the release of downstream IL-8 in a time-dependent manner, causing sustained inflammation and tumorigenesis [84]. OipA synergizes with CagA, similarly inducing pro-inflammatory signaling and IL-8 secretion in gastric epithelial cells. Additionally, this protein can trigger neutrophil infiltration and dendritic cell inhibition, thereby contributing to the pathogenesis of gastric cancer [85].

### *Porphyromonas gingivalis* (*P. gingivalis*)

*Porphyromonas gingivalis* is an inflammophilic microorganism that that absorbs these nutrients to sustain its growth while inducing a dysregulated inflammatory microenvironment, which is considered a tumorigenic factor. Prospective cohort studies have long suggested a correlation between the presence of the oral pathogen *P. gingivalis* and an increased cancer risk [86]. However, the precise mechanisms by which *P. gingivalis* contributes to carcinogenesis remain unclear. Recent research has revealed that the oral microbiota *P. gingivalis* can translocate to the pancreas and upregulate the expression of CXCR4 (C-X-C chemokine receptor type 4) in pancreatic cancer cells, a chemokine receptor capable of binding to the fimbriae of *P. gingivalis* [87]. Under environmental stress and chemotherapy, the upregulated CXCR4 further facilitates the colonization of *P. gingivalis* within cancer cells, thereby enhancing their survival. This phenomenon implies a mutualistic symbiotic relationship between *P. gingivalis* and pancreatic cancer cells [88]. Moreover, the highly enriched *P. gingivalis* in tumor tissues can activate the NLRP3 inflammasome and induce neutrophils to release elastase, thereby fostering inflammatory responses and shaping the suppression of the tumor immune microenvironment [89, 90].

Intratumoral *P. gingivalis* can also modulate macrophage activity. When macrophages are stimulated by *P. gingivalis* or its lipopolysaccharides, their secretion of IL-1α, CCL3, and CCL5 significantly increases, with different strains of *P. gingivalis* inducing varying levels of cytokine production [91, 92]. Additionally, *P. gingivalis* inhibits the phagocytic activity of macrophages towards cancer cells and promotes the polarization of M1-type macrophages to the M2 type. This ability of *P. gingivalis* to modulate macrophage function is partly dependent on the sphingolipid content in the bacterial membrane [93].

### Other intratumoral commensal bacterial

Beyond the above commonly implicated intratumoral bacteria driving tumor progression, emerging evidence has identified additional harmful microbial species. In colorectal cancer, *Eubacterium rectale* endotoxin exacerbates colitis and induces tumorigenesis by activating the transcription factor NF-κB in normal colonic epithelial cells [94]. Similarly, *Actinomyces* co-localizes with colorectal cancer-associated fibroblasts and reduces CD8^+^ T lymphocyte infiltration within the tumor microenvironment by activating the TLR2/NF-κB pathway, thereby promoting tumor progression [95]. In gastric cancer, *Methylobacterium* contributes to tumor development by downregulating TGFβ expression and decreasing the frequency of CD8^+^ tissue-resident memory T cells in the tumor [96]. In addition, the interplay between intratumoral commensal bacterial communities may result in a synergistic pro-tumor effect. In lung cancer, commensal bacteria trigger Myd88-dependent production of IL-1β and IL-23 from myeloid cells. This, in turn, induces the proliferation and activation of Vγ6^+^Vδ1^+^ γδT cells that produce IL-17 and other effector molecules, thus fostering tumor growth [97]. Distinct and abundant intratumoral commensal bacteria drive suppressive monocyte differentiation in pancreatic cancer through selective Toll-like receptor ligation, consequently inducing T-cell anergy and creating an immunosuppressive microenvironment [98].

The intricate interplay between the intratumoral microbiome and cancer progression represents a compelling avenue for investigation in contemporary research. These intratumoral microbiota, which play a crucial role in promoting an immunosuppressive microenvironment, are often traceable to the oral cavity or gastrointestinal tract, thereby providing a clue to the origins of intratumoral microbiome. The close interaction between intratumoral microbiome and immune cells has catalyzed the exploration of its role within the tumor microenvironment. Beyond tumor-specific bacteria, a myriad of bacterial species inhabits tumors independently or as commensal residents, orchestrating immune responses that contribute to pro-tumorigenic effects. It is imperative to note, however, that the role of intratumoral bacteria is a topic rife with controversy. Not all intratumoral bacteria fit the archetype of tumor-promoting “malignant collaborators”; some emerge as “protective allies” actively supporting anti-tumor immunity, a subject that will be expounded upon in the ensuing section.

### Anti-tumor intratumoral microbiota: the protective allies

Although the majority of studies emphasize the negative regulatory role played by the intratumoral microbiome on the host, akin to the dual nature inherent in all things, there are also a part of intratumoral bacteria that can exert an anti-tumor effect through positive immunomodulation.

### *Akkermansia muciniphila* (*A. muciniphila*)

*Akkermansia muciniphila* is a gut probiotic endowed with a myriad of potent functions, including weight loss, anti-aging, inhibition of neurodegenerative diseases, and prediction of immunotherapy efficacy [99]. Recent research has revealed that this probiotic also resides within the tumor microenvironment, where it can remodel the immune milieu to inhibit inflammation-associated tumorigenesis. In CRC, *A. muciniphila* suppresses tumor growth by inducing the activation of the TLR2/NF-κB/NLRP3 pathway and promoting the accumulation of M1 macrophages [100]. Furthermore, the membrane protein Amuc_1100 of *A. muciniphila* has been demonstrated to enhance gut barrier function and reduce inflammation through TLR2 signaling, while also inducing the production of tumor cytotoxic cytokines such as IFN-γ and granzyme B to inhibit tumorigenesis [101]. Another specific membrane protein of *A. muciniphila*, acetyltransferase, has been shown to reprogram the tumor microenvironment through immunomodulation, thereby reducing the incidence of CRC. Specifically, during tumorigenesis, the acetyltransferase of *A. muciniphila* enters cancer cells via macropinocytosis and induces the transcription and secretion of HSP70 through H3K14ac, accelerating tumor-specific T cell responses and promoting protective immunity [102]. The beneficial effects of *A. muciniphila* in the gut have been well substantiated, and it also appears to play a cancer-inhibiting role in the tumor microenvironment, albeit requiring further investigation to confirm these findings.

### *Lactobacillus*

Intratumoral *Lactobacillus* can also bolster anti-cancer efficacy through immune modulation. In breast cancer, the probiotic *Lactobacillus plantarum* promotes butyrate production, which enhances butyryl-coenzyme A transferase activity, suppresses inflammation, and fosters the formation of a healthy microbiota, thereby inhibiting tumor growth [103]. *Lactobacillus reuteri (L. reuteri)*, persisting in melanoma, enhances the efficacy of antitumor immunity by secreting the dietary tryptophan catabolite I3A (Indole-3-acetic acid), which stimulates CD8^+^ T cells to produce IFN-γ. Additionally, the antitumor immune response of *L. reuteri* can be further amplified by a tryptophan-rich diet [104]. Moreover, *Lactobacillus johnsonii* (*L. johnsonii*) can facilitate the production of indole-3-propionic acid (IPA), which modulates the stemness program of CD8^+^ T cells and promotes the proliferation of exhausted CD8^+^ T cells, ultimately enhancing pan-cancer responsiveness to immune checkpoint inhibitor (ICI) therapy [105]. The identification of intratumoral *Lactobacillus* and elucidation of their mechanisms provide a more rational basis for designing novel dietary and probiotic combination therapies for cancer patients.

### *Clostridium*

*Clostridium* may also serve as a good prognostic marker in the tumor. High levels of intratumoral *Clostridium* are significantly associated with reduced CCL2 expression and downregulated PI3K activity, which might lead to a decrease in the recruitment of myeloid-derived suppressor cells (MDSCs) to the tumor microenvironment. This, in turn, could result in an increased infiltration of CD8^+^ T cells in bile tract cancers [106]. Furthermore, *Clostridium butyricum (C. butyricum)*, a commensal bacterium within the digestive system, exerts its probiotic effects primarily through the production of short-chain fatty acids (SCFAs) [107]. In oncological contexts, *C. butyricum* inhibits cancer cell proliferation predominantly by interacting with the Wnt/β-catenin signaling pathway to reduce bile acids and increase SCFAs production [108]. Another species, *Clostridium sporogenes*, augments the efficacy of CD8^+^ T cell-mediated immunotherapy by assisting other probiotics in elevating levels of IPA in the bloodstream [105].

### Other intratumoral commensal bacterial

In addition to the previously discussed tumor-specific probiotics, numerous other commensal bacteria exhibit anti-cancer properties through immune mechanisms, either as isolated species or as part of a consortium. To elucidate the intricate interactions among metabolites, immune cells, and microbial composition within the tumor microenvironment and their relationship to the progression of CRC, Xusheng Zhang et al. performed a comprehensive analysis of the commensal microbiota in normal colonic tissue, adjacent non-tumorous tissue, and tumor tissue from CRC patients. Their investigation revealed that bacteria of the *Lachnospiraceae* family, specifically *Ruminococcus gnavus* and *Blautia producta*, colonizing tumors can rapidly degrade lysophosphatidylcholine, thereby maintaining the tumor immune surveillance function of CD8^+^ T cells and playing a pivotal role in preventing colorectal cancer development [109]. Furthermore, recent research by Ghaddar B et al. has demonstrated that intratumoral bacterial communities can induce immune cell infiltration and potentiate anti-tumor responses by enhancing programmed cell death protein 1 (PD-1) signaling and responses to intracellular infection. This process also involves the downregulation of FOXO-mediated transcription and interferon-gamma signaling, which inhibits regulatory T cell activation [46]. Additionally, the peptidoglycan of gram-negative bacteria engages the nucleotide-binding oligomerization domain-containing protein 1 (NOD1) to activate the CCR6-CCL20 axis, consequently upregulating isolated lymphoid follicles (ILFs) [110]. Further research is imperative to substantiate the anti-tumor effects of these bacteria, thereby supporting their potential use as therapeutic probiotics.

**Fig. 2:**
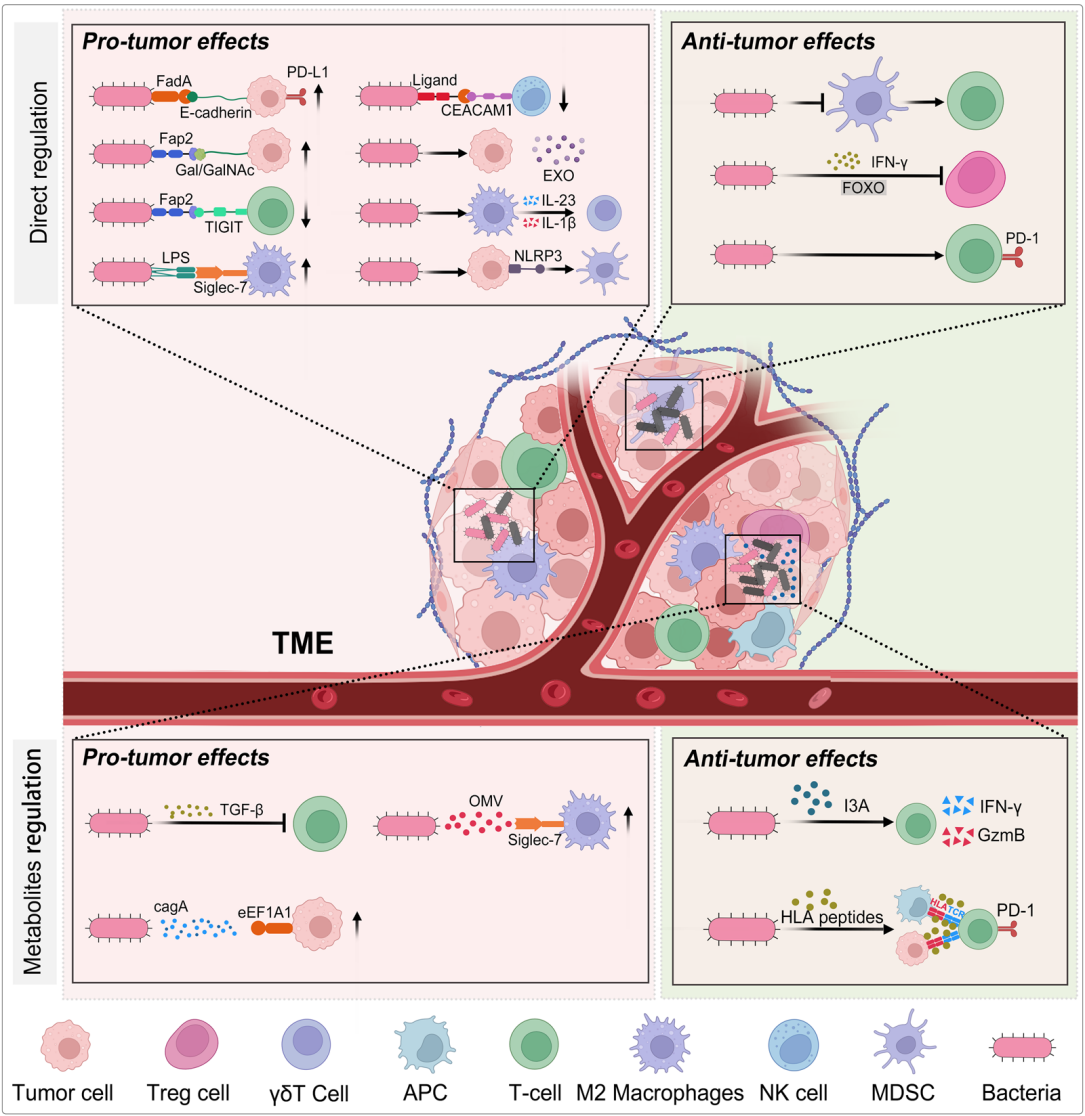
Intratumoral microbiome exert pro-tumor and anti-tumor effects through immune regulation

This body of evidence highlights the complex and multifaceted interactions between intratumoral bacteria and host immune responses, revealing differences in the influence of intratumoral bacteria on tumors. These discrepancies are attributable not only to the inherent characteristics of the bacteria but also to differences in technological platforms and sample processing of the intratumoral microbiome, which is also one of the problems faced by current research on tumor microbiomes. There is a need to develop new techniques that are more suitable for the detection of trace tumor microbiomes and to develop standard procedures for processing tumor microbiome specimens. Understanding the crosstalk among microbes, the immune system, and cancer cells in the tumor microenvironment may provide new therapeutic intervention strategies for cancer treatment. Targeting the intratumoral microbiota and its immunomodulatory effects may improve patient outcomes and alter the trajectory of this devastating disease (Fig. 2).

## Intratumoral microbiome influence therapeutic response through immunomodulation

### Impact of the intratumoral microbiome on immunotherapy response

Cancer cells are cunning and evade immune surveillance by overexpressing immune checkpoints and recruiting immunosuppressive cells, thus creating a more favorable environment for their growth and development [111]. ICI therapy, which activates anti-tumor immune responses by inhibiting the interactions between T-cell inhibitory receptors and their cognate ligands, is one of the most frequently employed clinical immunotherapeutic approaches. However, ICI therapy is associated with significant individual variability and low response rates, with less than 20% of the patients benefiting from it [112]. Hence, identifying the factors influencing the efficacy of immunotherapy is essential to enhance its effectiveness and broaden the scope of its beneficiaries, thereby providing a new avenue for improving immunotherapeutic outcomes.

In recent years, with the growing interest in tumor microbiome research, the impact of intratumoral bacteria on immunotherapy has gradually been unveiled. Recent studies have found that bacteria in the tumor microenvironment can influence the response to ICI therapy by mediating T cell immune responses. Kalaora et al*.* discovered HLA peptides from bacteria in melanoma that can be co-presented by antigen-presenting cells and tumor cells, thereby increasing the possibility of presenting more immunogenic antigens, promoting T cell activation, and consequently enhancing the benefits of ICI therapy [17]. The facilitative role of the intratumoral microbiota in melanoma immunotherapy was further demonstrated in preclinical models treated with probiotics. Specifically, the study found that *L. reuteri* within melanoma mouse model promotes an immune-stimulatory tumor microenvironment through the release of dietary tryptophan catabolite I3A, which promotes the infiltration of IFNγ-producing CD8^+^ T cells mediated by AhR signaling, thereby enhancing the response to PD-L1 therapy in patients [104]. Additionally, during ICI treatment, *F. nucleatum* was found to increase the accumulation of IFN-γ^+^CD8^+^ T cells in CRC patients, thereby enhancing the cancer's sensitivity to anti-PD-L1 therapies [113]. The tumor microbiome can also directly affect the effectiveness of ICI treatment by regulating immune checkpoints. In oral squamous cell carcinoma and CRC, intratumoral bacteria stimulate MAPK, NF-κB, and other signaling pathways through microbial-associated molecular patterns, promoting the formation of an immunosuppressive microenvironment by enhancing the production of MDSCs and neutrophils. In this immunosuppressive microenvironment, the immune checkpoints CTLA4 and PD-1 are upregulated, and regions with a high bacterial load in the tumor form a barrier that hinders the infiltration of T cells into the tumor [14]. Furthermore, intratumoral *gammaproteobacteria* seem to downregulate the expression of PD-L1 and lead to poor responses to ICI therapy in patients with non-small-cell lung cancer [114].

### Impact of the intratumoral microbiome on chemotherapy response

Although conventional chemotherapy is not traditionally classified as an immunotherapy, it is essential to acknowledge that its efficacy relies on intact immune responses. This observation underscores the hypothesis that the composition of the intratumoral microbiota plays a pivotal role in modulating an individual's response to these therapeutic modalities. This has been demonstrated in several studies on cancer chemotherapy. In CRC patients receiving standard 5-FU-based adjuvant chemotherapy after curative surgery, *F. nucleatum* infection triggered significant upregulation of the BIRC3 gene, which was mediated through the TLR4/NF-κB pathway. Consequently, this infection reduces the sensitivity of CRC cells to 5-FU chemotherapy, culminating in the development of drug resistance [115]. Inflammasome-mediated inflammation plays a pivotal role in innate immunity [116]. As a member of the inflammatory complex family, NLRP3 acts as a scout for monitoring and recognizing various danger signals, such as pathogenic bacteria and their metabolites. This subsequently induces an inflammatory response that promotes tumor growth and chemoresistance [117, 118]. In esophageal squamous carcinoma, *F. nucleatum* enriches MDSCs by inducing high expression of NLRP3, which results in cisplatin resistance [119]. The inflammatory response usually causes immunosuppression, which weakens the therapeutic response [120]. Neoadjuvant chemoimmunotherapy (NACI) has become the first-line treatment option for gastroesophageal cancer, with the role of the intratumoral microbiome being explored in this context. In patients with esophageal squamous cell carcinoma who received NACI, the abundance of *Streptococcus* in tumor tissue was significantly higher in responders than in non-responders and was predictive of prolonged disease-free survival (DFS). Further analysis of immune cell infiltration revealed an increasing trend of GrzB^+^ and CD8^+^ T-cell infiltration in patients with high intratumoral streptococcal abundance [15], suggesting that intratumoral bacteria play an important role in chemotherapy combined with immunotherapy.

Overall, the available evidence shows that the intratumoral bacteria related to immunotherapy responses belong to different taxa. Contradictory roles also exist for the same bacterial genus within the same cancer (increasing ICI benefits/promoting resistance) [113, 115]. The reasons behind these contentious findings primarily relate to the technical platforms and sample handling processes. One major challenge in intratumoral microbiota research is sample contamination, which can lead to misinterpretation of microbial composition and function [10]. Additionally, since bacterial metabolites play a key role in influencing the host, their production is highly dynamic, varying with time and environmental conditions. Consequently, factors such as sampling methods, handling procedures, and storage conditions—including time, temperature, and preservation techniques—can significantly impact the accuracy and reproducibility of metabolite detection [31]. However, the impact of the tumor microbiome on treatment response is indisputable. These data provide compelling evidence that intratumoral microbiota can modulate tumor immunity and respond to immunotherapy.

## Intratumoral microbiome influence tumor prognosis through immunomodulation

Intratumoral microbiomes may affect proximal and distant immunity and alter the clinical outcomes of patients with tumors [121, 122]. There is a significant correlation between intratumoral bacterial load and cellularity of CD8^+^ T cells, NK cells, PU.1^+^ macrophages and CD66B^+^ neutrophils, the higher the bacterial load; the fewer the immune stimulation cells, which significantly reduces the survival rate of cancer patients [94, 96, 123, 124]. In contrast, pancreatic cancer long-term survivors exhibit a more diverse and abundant intratumoral microbiome, along with higher frequencies of CD4^+^ and CD8^+^ T cells, compared to short-term survivors [125]. In addition, high expression of CD206 (a marker for M2 macrophages) in the tumor stroma is associated with an increased burden of gram-positive bacteria in the tumor and predicts poor prognosis for esophageal squamous cell carcinoma [126].

Tumor metastasis and recurrence are major challenges for tumor treatment. Compared with early-diagnosed tumors, metastasis and relapse make treatment more difficult. Emerging evidence highlights the contribution of intratumoral microbiota to tumor metastasis. In a pan-cancer metastasis cohort, the DNA of *F. nucleatum* can be widely detected in tumors, and its high abundance in non-small cell lung cancer is indicative of poorer outcomes [127]. The abundance of *F. nucleatum* is also strongly associated with an increased density of myeloid-derived suppressor cells (MDSCs), and can promote macrophage infiltration by activating CCL20 and inducing M2 polarization of macrophages to enhance CRC metastasis [128, 129]. Additionally, *F. nucleatum* selectively acquires miR-155-5p and miR-205-5p by activating Myd88-dependent TLR4-mediated signaling pathways to inhibit ADH1B and TGFBR2 expression, thereby promoting the migration of laryngeal cancer cells to the lungs and shortening the disease-free survival of patients [130]. Furthermore, the surface adhesin Fap2 of *F. nucleatum* can induce the secretion of pro-inflammatory cytokines IL-8 and C-X-C motif chemokine ligand 1 (CXCL1) and NOD-like receptor family pyrin domain containing 3 (NLRP3) activation to promote tumor migration and invasion [131]. Additionally, exosomes can serve as mediators through which intratumoral bacteria facilitate metastasis. Studies have revealed that tumor cells infected with bacteria may augment exosome secretion to establish an immunosuppressive microenvironment [78, 132, 133]. Notably, *F. nucleatum* infection in tumor cells stimulates the secretion of exosomes enriched with miR-1246, miR-92b-3p, miR-27a-3p, and CXCL16, RhoA, and IL-8 [134]. These exosomes serve as mediators of intercellular communication, transferring their molecular cargo to uninfected cells, including but not limited to tumor cells [134, 135], immune cells [136–138], epithelial cells [139], fibroblasts [140, 141] and endothelial cells [142]. This cascade ultimately facilitates the establishment of a pre-metastatic niche characterized by immunosuppression, inflammation, angiogenesis, vascular permeability, lymphangiogenesis, organotropism, and cellular reprogramming, all of which contribute to tumor cell colonization and metastasis [143].

The intratumoral microbiome can also influence tumor recurrence through immune modulation. In CRC, intratumoral bacteria sustain the activation of the NFκB-TNF-α-IL-6 pathway to promote the activation of metalloproteins and colony-stimulating factor 1–3 (CSF1-3) to result in an increased risk of relapse [144, 145], and also increase the mortality by reducing tumor-infiltrating lymphocytes infiltration [146]. Similarly, the commensal microbiota in breast cancer tissue can also interact with host immunity to influence the risk of local recurrence [147]. Remarkably, *F. nucleatum*, previously regarded as a tumor-promoting intratumoral bacterium, has been found to reduce the recurrence rate of oral squamous cell carcinoma and extend patient survival, which seems to be associated with fewer M2 macrophages, CD4 lymphocytes, fibroblasts, and TLR4, and increased TNFSF9 and IL-1β [148]. These findings provide new clues for using the intratumoral microbiome as a biomarker to predict survival.

## Modulating intratumoral microbiome for enhancing cancer therapy

**Fig. 3:**
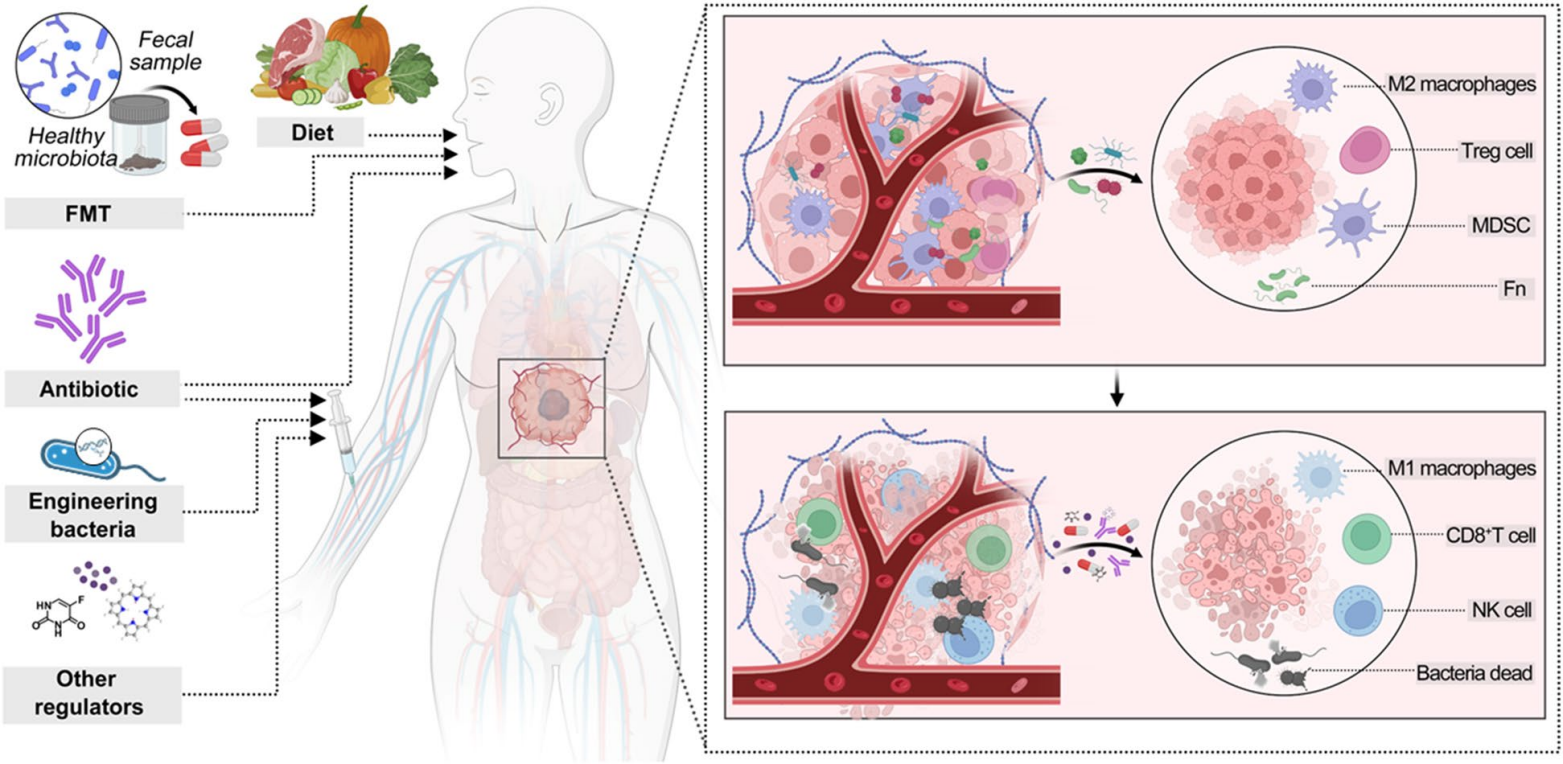
Modulating intratumoral microbiome for enhancing cancer therapy

While current research on manipulating the tumor microbiome to improve treatment is still in the exploratory stages, existing evidence suggests that the tumor microbiome can exert beneficial or detrimental effects on host physiology by modulating tumor immunity. In this context, enhancing anticancer immunity through the modulation of the intratumoral microbiota is a viable treatment option for patients with cancer (Fig. 3).

### Antibiotics targeting harmful intratumoral bacteria

Antibiotics are powerful tools that are used against bacteria. A study published in *Science* in 2017 highlighted a significant reduction in the intratumoral *F. nucleatum* burden and cancer cell proliferation, along with decreased metastatic risk in *F. nucleatum*-positive CRC mouse models upon treatment with metronidazole [149]. In another study, ciprofloxacin and gemcitabine were delivered into colorectal tumors when loaded into HA. Leveraging the acidic pH and hyaluronidase response, both agents were released into the tumor microenvironment, leading to the dual action of killing intratumoral bacteria and cancer cells. Notably, the destruction of bacteria by this method facilitates dendritic cell maturation, whereas the released gemcitabine inhibits MDSCs, thereby fostering T cell-mediated anti-tumor immunity to augment immunotherapy [150]. These results imply that antibiotic-based interventions targeting intratumoral microbiota could serve as potential avenues to bolster cancer treatment. Nevertheless, the broad-spectrum antibacterial nature of metronidazole and ciprofloxacin can also target beneficial constituents of the tumor microbiome, potentially compromising microbial diversity and impeding cancer immunotherapy [151]. To address this challenge, Wang et al*.* assessed the anaerobic activation properties of nitroimidazoles and devised an antibiotic silver-tinidazole complex enclosed within liposomes. This compound specifically targeted the tumor-promoting anaerobic bacterium *F. nucleatum*, eliciting the discharge of cancer-specific microbial neoantigens and fostering augmented infiltration of CD8^+^ T cells. Employing this approach to eliminate tumor-promoting bacteria has the potential to convert an immune-cold tumor into an immune-hot one, thereby activating the immune system to recognize tumor cells [152].

Adjunctive use of antibiotics in cancer treatment can effectively reduce intratumoral pathogenic bacteria, thereby alleviating tumor progression, enhancing immunotherapy efficacy, and overcoming tumor resistance induced by harmful microbes. Compared to other microbiome modulation strategies, antibiotic intervention is relatively simple and can be monitored in real-time through conventional detection methods [153]. However, the use of antibiotics also has limitations, such as their non-selective impact on the host microbiome, which may trigger adverse reactions, disrupt the normal microbiota, and promote the emergence of resistant strains [154]. Additionally, their application in cancer is limited to the depletion and prevention of known carcinogenic microorganisms [155]. Therefore, careful selection and prudent use of antibiotics are essential in clinical practice to avoid overuse.

### Reshaping the intratumoral microbiota landscape through fecal microbiota transplantation (FMT)

It is well established that FMT directly modulates the gut microbiota, thereby influencing cancer treatment. Recent studies have suggested that the post-FMT gut microbiota can migrate via the bloodstream and lymphatic pathways, colonize the tumor microenvironment, and exert immunomodulatory effects [104, 125]. In the case of pancreatic cancer, individuals with long-term survival demonstrate greater diversity and abundance of their intratumoral microbiomes, along with higher frequencies of CD4^+^ and CD8^+^ T cells, than short-term survivors. Notably, the transplantation of fecal microbiota from long-term survivors to antibiotic-treated mice resulted in the detection of human donor bacteria within the tumor tissues of recipient mice, effectively controlling tumor progression by fostering increased immune cell infiltration [125]. This finding was corroborated by a recent study wherein antibiotic-treated mouse models showed significantly smaller tumors in mice that received FMT from responders than in those from non-responders under ICI therapy. Tracing the origin of intratumoral bacteria revealed the presence of human donor-derived bacteria in the tumor tissues of FMT-treated mice. Further investigation of their impact on host immunity indicated their capacity to induce anti-tumor effects by enhancing CD8^+^ T cell infiltration and reducing the number of Treg cells [104].

Currently, FMT is most established in the treatment of *Clostridium difficile* infections, with success rates reaching up to 95%, and it has been incorporated into clinical guidelines [156, 157]. In the field of oncology, both preclinical and clinical studies suggest that FMT may enhance the efficacy of ICIs by modulating the microbiome [158]. The alteration of the microbiome profile in FMT recipients may enhance the therapeutic activity of FMT through competitive growth [159, 160]. In addition, FMT is considered a milder approach compared to other treatments and may reduce the toxicity of chemotherapy and immunotherapy drugs, thereby alleviating radiation-induced intestinal damage and decreasing immune-related adverse events (irAEs) [161]. However, FMT may encounter limitations in certain scenarios, including when resistance is independent of the microbiome, when donor-recipient microbiota compatibility is lacking, or when pathogens are inadvertently transplanted into immunocompromised cancer patients [162]. Such factors can result in treatment failure, the exacerbation of irAEs, or the emergence of secondary infections, which further complicate the clinical management of cancer. To mitigate these risks, personalized microbiome profiling is essential to ensure compatibility between the donor and recipient, alongside rigorous pathogen screening to safeguard against potential infections. While FMT holds considerable promise in reshaping the tumor microbiome and modulating tumor immunity, careful selection of an optimal donor is critical to avoiding these pitfalls and maximizing its therapeutic efficacy.

### Dietary regulation

Prompted by the regulatory impact of diet on gut microbiota, multiple research teams have initiated investigations into the mediation of the regulatory role of intratumoral microbiota in cancer through diet. In a double-blind placebo-controlled clinical trial, the continuous administration of fish oil in the month preceding tumor resection among patients with breast cancer was found to significantly modify the composition of the tumor tissue microbiota [163]. However, this study did not investigate the implications of these intratumoral microbial changes on tumor development and cancer treatment. In a recent study, researchers attempted to enhance immunotherapy by modulating the intratumoral probiotic *L. reuteri* through dietary regimens. They subjected mice to either a high- or low-tryptophan diet before implanting tumor cells into the *L. reuteri*-treated mouse model and maintained this dietary protocol during ICI therapy. These findings highlight that a diet rich in tryptophan substantially bolsters the *L. reuteri*-mediated anti-tumor effect. Further investigation revealed that the beneficial impact on the tumor promoted by the high tryptophan diet was mediated through the augmentation of AhR activity in the TME [104].

Dietary modulation is an appealing approach to shaping the host microbiome towards a healthier microbial ecological balance. Leveraging individual dietary responses in the context of cancer could allow for personalized diet plans that not only prevent or treat cancer and its complications but also optimize treatment outcomes [164]. Dietary interventions can be considered one of the most cost-effective, safest, and simplest adjuncts to cancer treatment [121]. However, most existing studies are based on preclinical models and often overlook the interindividual variability in human physiological and disease responses [164]. Moreover, the complexity of cancer treatment regimens makes it challenging to maintain the balance of beneficial microbial communities, necessitating professional dietary counseling and guidance [121]. These limitations could hinder the generalizability of dietary interventions across diverse patient populations, potentially reducing their effectiveness and leading to suboptimal outcomes in clinical settings. Therefore, future research should include more human-based clinical trials that account for individual physiological and disease-related variations, tailoring dietary interventions and treatment strategies to the specific microbiome and metabolic responses of each patient.

### Engineered probiotics for modulating the intratumoral microbiome

The advancement of synthetic biology and genetic engineering has led to the development of probiotic therapeutics with high tolerance, high targeting and high loading capacity. To improve cancer treatment outcomes, the administration of engineered probiotics to modify or reconstruct the intratumoral microbiome and TME disrupted by tumor cells has emerged as a novel therapeutic approach. Currently, the hypoxic environment associated with tumor growth has been identified as a potential therapeutic target. Building on this, Zi-Yi Han et al. developed an engineered probiotic therapeutic agent, LGG@Ga-poly, which utilizes a Ga-polyphenol network and chitosan nanocoating to modify *Lactobacillus rhamnosus*. This agent selectively targets PDA tumors, suppresses intratumoral microbiota, and enhances T cell recruitment, thereby reversing the immunosuppressive TME and improving the efficacy of ICI therapy [165]. In addition to enhancing cancer treatment, the use of engineered probiotics to regulate the intratumoral microbiome for cancer prevention is also a potential strategy. Ankan Choudhury et al. employed gene editing techniques to design *Lactococcus lactis* engineered to secrete antimicrobial peptides (gAMPs) targeting *H. pylori*, which efficiently eradicated *H. pylori* within five days, thus preventing gastric cancer [166]. Emerging strategies involving engineered probiotic mimics also show promise. Qian Chen’s team developed a nanodrug mimicking *F. nucleatum* by fusing liposomes loaded with the antibiotic colistin with the cytoplasmic membrane of *F. nucleatum*. These liposomes, which express Fap-2 on their surface, specifically target colorectal tumors overexpressing Gal-GalNAc and accumulate in the TME. This engineered mimic selectively eradicates *F. nucleatum* residing in the tumor, overcoming immune therapy resistance induced by this pathogen [167].

While the elimination of harmful intratumoral bacteria can enhance anti-tumor efficacy, the question remains whether the preservation of beneficial intratumoral bacteria could also promote cancer therapy. Existing studies have provided an answer. Zheng et al. found that abundant *Streptococcus* within tumors of patients with oral squamous cell carcinoma can activate immune responses and is associated with a better prognosis [168]. To leverage this finding, they combined a viscous hydrogel containing silver nanoparticles with *Streptococcus* to promote its proliferation while suppressing the growth of other intratumoral bacteria. When this formulation was combined with ICI therapy, it resulted in increased infiltration of CD8^+^ T cells and significantly enhanced the efficacy of immunotherapy [168].

Engineered probiotics possess tremendous potential as alternative therapeutic agents to genetically modified biopharmaceuticals such as monoclonal antibodies and therapeutic proteins. The former can overcome the limitations of high production costs, short half-lives, and delivery restrictions associated with the latter by secreting therapeutic proteins, delivering antigens, clearing pathogens, and modulating the immune system [169, 170]. However, the safety of engineered probiotics remains a significant concern. There is a risk of gene leakage during the genetic modification process, and engineered probiotics may induce allergic reactions and inflammation in individuals with weakened immune systems [171]. Therefore, in the future development of engineered probiotics, researchers must select appropriate genetic tools and design precise molecular switches to control and monitor the effects of these probiotics. Furthermore, a comprehensive understanding of the synthetic biology, heterologous expression, and secreted substances of the selected probiotics, along with strict regulation of bacterial dosage, is essential to balance therapeutic efficacy and bacterial toxicity.

### Other intratumoral microbiota regulators

The small molecule inhibitor 5-FU, commonly used in anti-cancer treatment, demonstrates potent antibacterial activity against intratumoral *F. nucleatum*, enhancing the efficacy of chemotherapy by inhibiting the growth of this oncogenic bacteria within the tumor. In the context of CRC, where 5-FU serves as a first-line chemotherapy agent, its superior anti-tumor effects in CRC when compared to that in other cancers may be partially attributed to its inhibitory influence on major oncogenic microbes within the tumor while killing cancer cells. Additionally, this research highlights the ability of CRC intratumoral *H. pylori* to metabolize 5-FU, thus shielding *F. nucleatum* and CRC cells from 5-FU-induced harm [172]. The administration of antibiotics concurrently to eliminate this protective effect of intratumoral *H. pylori* could represent a viable strategy. Moreover, antibacterial metalloporphyrins also demonstrate notable inhibitory effects on intratumoral bacteria. Qu et al*.* devised an antibacterial nanoplatform loaded with gold nanoparticles utilizing fetal bovine serum albumin as a carrier and incorporating an antibacterial metalloporphyrin sonosensitizer. This platform effectively suppresses *F. nucleatum* in CRC tumors and reduces the levels of anti-apoptotic proteins in cancer cells, thereby enhancing the efficacy of sonodynamic therapy. Furthermore, this antibacterial platform mitigates the generation of light-induced reactive oxygen species (ROS), thereby mitigating the inflammation and skin damage associated with antibacterial metalloporphyrins [173]. Another strategy based on protein-supported copper single-atom nanozymes targets both *F. nucleatum* and cancer cells by generating ROS and Glutathione (GSH), thereby achieving destruction of pathogen-tumor symbionts [174]. Moreover, the tumor microbiota is modulated by Col1 homotrimer, an aberrant homo-trimeric variant with specific carcinogenic properties produced by pancreatic cancer cells. Its absence induces a beneficial alteration in the intratumoral microbial landscape, characterized by reduced *Bacteroidales* and increased *Campylobacterales*. This shift is associated with decreased MDSCs and increased T cells, thereby enhancing the efficacy of anti-PD-1 therapy. Notably, the use of broad-spectrum antibiotics abolishes this change, demonstrating the potential to enhance immunotherapy effectiveness by regulating the tumor microbiota through Col1 homotrimer [175]. These intratumoral microbiota modulators expand the avenues for manipulating the microbiome to improve cancer treatment. Further research is needed to explore their feasibility and value, contributing to the accumulation of more comprehensive evidence for future clinical translation.

In summary, strategies to enhance cancer treatment by modulating the intratumoral microbiota, including antibiotics, FMT, dietary interventions, and engineered probiotics, represent promising avenues for the future. These approaches aim to regulate the tumor microenvironment by eradicating or reshaping the intratumoral microbial landscape, thereby promoting antitumor immunity. They offer innovative pathways to improve therapeutic efficacy and have the potential to address critical challenges associated with conventional treatments, including off-target effects, drug resistance, and low response rates, underscoring their significant clinical translation potential. However, bridging the gap between laboratory discoveries and clinical implementation remains a formidable challenge. Currently, clinical trials for therapies targeting intratumoral microbiota are scarce, with lingering concerns about their safety and the need for enhanced efficacy. Given that intratumoral bacteria are not represented by a single species but rather by a diverse microbial community collectively influencing tumor behavior, the inherent heterogeneity among individuals complicates research and therapeutic development. Before any treatment recommendations can be made for cancer patients or individuals at risk of cancer, further clinical studies are required to validate the safety and efficacy of these therapeutic strategies. Moreover, due to tumor heterogeneity, single-agent therapies often fall short of achieving complete tumor remission. Therefore, combining strategies that regulate intratumoral microbiota with traditional treatments such as chemotherapy, radiotherapy, and immunotherapy holds promise for achieving superior clinical outcomes. This integrated approach represents a critical direction for future clinical trials and a potential breakthrough in cancer treatment.

## Challenges and prospects

The complete elucidation of intratumoral microbiome-mediated immunomodulatory mechanisms and the precise identification of immunomodulatory intratumoral microbiome strains will help elucidate the effects of intratumoral microbiomes on tumor development and metastasis, improve the efficacy of tumor therapies, monitor tumor responses to therapies, and improve tumor prognosis. Although tremendous efforts have been made to achieve outstanding advances in this research field, further investigations still face the following challenges. First, the bacterial biomass in the tumor specimen was low; therefore, the bacterial genomic content was likely overwhelmed by the host/tumor genomic content, resulting in low PCR efficiency and inaccurate results [176]. Therefore, using commercial kits such as LYPMA and QIAamp, which can deplete the host/tumor genome, is the key to increasing the efficiency of intratumoral microbiome detection. Secondly, sample contamination is a serious issue. Sample contamination can occur during sampling, library preparation, or the utilization of detection kits [177]. This causes the data of the target intratumoral microbiome to be masked by contaminating bacteria, particularly when PCR amplifies the contaminant data. Newly developed bioinformatics programs, such as SourceTracker and Decontam, can be used to remove pollutant taxa [178, 179]. Third, the intratumoral microbiome and tumor cells may have co-evolved. Tumor cells constantly evolve to endure environmental pressures such as hypoxia and anti-tumor agents. When antibiotics are used to manipulate the microbiome during immunotherapy, tumor cells may still undergo molecular evolution (for example, stress-induced mutagenesis). The microbiome may undergo a similar evolution in the stressful tumor microenvironment [180, 181], and this co-evolution presents a more complex and refined challenge to deciphering the role of the intratumoral microbiome in tumors.

Understanding the ability of microbes to colonize the tumor microenvironment and interact with immune cells could contribute to the development of vaccines, targeted intratumoral microbiome therapies, and engineered probiotics. The malignant crosstalk between tumors and the intratumoral harmful microbiota creates a vicious cycle within the tumor microenvironment, and antigen-based vaccine delivery targeting the microbiota can significantly inhibit tumor-associated microorganisms. During this process, the host generates robust systemic and mucosal antibody responses, along with cell-mediated immunity, which provide long-lasting protection and enable rapid recall upon subsequent exposure to homologous antigens. These responses typically involve the activation of T cell memory and effector subsets, as well as the induction of long-lived memory B cells [1]. Vaccine efficacy is typically measured by the quantity of antigen-specific antibodies induced by the vaccine [2]. However, focusing solely on antibodies overlooks the complexity of the immune system, which has evolved multiple defense mechanisms, including T cell responses and innate immunity to pathogens. For example, the presence of varicella-specific T cells, rather than just antibodies, is considered the best indicator of vaccine effectiveness [4]. The balance of multiple immune mechanisms, such as the combined evaluation of CD8^+^ T cell frequency and antibody titers, may synergistically contribute to the recognition, control, and elimination of pathogens. Therefore, identifying the precise immune mechanisms associated with disease protection can provide valuable insights for designing more effective next-generation vaccines.

The core of antitumor immunity lies in the ability of immune cells to recognize and eliminate tumor-associated antigens. Bacteria, with their complex chemical composition and diverse antigens, can be recognized by the host immune system and trigger immune responses [182]. Leveraging this property, bacteria can potentially produce or deliver tumor-associated antigens, stimulating specific and durable antitumor adaptive immune responses. This offers new avenues for developing targeted therapies against intratumoral microbiota and engineering probiotic-based strategies. In the realm of intratumoral microbiota-targeted therapies, bacteria within tumor and immune cells provide a novel source of antigenic epitopes. Eliminating intracellular bacteria can expose these microbial-specific antigenic epitopes, generating new cancer-associated antigens and fueling antitumor immune responses [152]. One common approach to selectively eradicate intratumoral bacteria involves antibiotics. For example, liposome-encapsulated silver-metronidazole effectively eradicates *F. nucleatum* within tumors, releasing novel cancer-specific microbial antigens and promoting CD8^+^ cytotoxic T-cell infiltration [152]. Moreover, it has been demonstrated that bacterial-induced T-cell responses can enhance antitumor immunity through cross-reactivity with MHC-I antigens in tumor cells, highlighting the ability of the microbiota to elicit specific T-cell responses [17]. Thus, both the heterologous epitopes of bacteria and their homologous epitopes shared with the host contribute to enhanced immunogenicity, facilitating T-cell recognition of tumor cells and thereby amplifying tumor suppression. In the context of engineered bacterial therapeutic strategies, bacteria serve as advantageous vectors due to their capacity to activate the innate immune system, particularly macrophages and NK cells [183]. TAMs are key players in the immunosuppressive tumor microenvironment, exhibiting antigen-presenting and immunosuppressive properties that limit the efficacy of immunotherapies. It is well-established that TAMs exist in two polarized states: the antitumorigenic M1 phenotype and the protumorigenic M2 phenotype [184]. Engineered bacteria, such as nanoparticle-modified *E.coli*, can promote the polarization of M2 macrophages into the M1 phenotype and activate innate immune responses in vivo, thereby enhancing antitumor immunity [185]. Additionally, NK cells, as critical components of innate immunity, can recognize and lyse a variety of target cells. Engineered *Salmonella* strains have been shown to activate NK cells through the production of IFN-γ, which, via self-regulatory feedback loops, promotes NK cells aggregation, activation, and cytotoxicity, achieving significant tumor suppression [186].Targeted elimination of intratumoral microbiota and engineered probiotic-based therapies effectively activate and regulate antitumor immune responses, highlighting the pivotal role of the immune system while offering innovative strategies for cancer treatment.

In contrast to its established role in cancer treatment, the application of the intratumoral microbiome in cancer diagnosis remains a significant challenge. The intratumoral microbiome may confer distinct tumor-specific features, which could facilitate personalized and precise diagnostic approaches. In pancreatic cancer, a multi-bacterial scoring model based on 12 significantly enriched dominant bacterial species has shown robust and consistent diagnostic performance, enabling reliable differentiation between pancreatic ductal adenocarcinoma (PDAC) and other pancreatic tumor types. This model consistently achieved an AUC value exceeding 0.8 across training, test, and external validation cohorts [187]. Similarly, microbiome-based diagnostics have demonstrated strong discriminatory power in central lung cancer. Salvador Bello et al. identified a significant enrichment of *Streptococcus* in the bronchial tissues of patients with central lung cancer. By using *Streptococcus* abundance as a diagnostic marker, they achieved sensitivity and specificity of 90.9% and 83.3%, respectively, in distinguishing central lung cancer patients from healthy controls [188]. In the future, utilizing microbiome-based features to identify tumors lacking any known oncogenic mutations may hold significant potential for cancer screening and diagnosis.

Furthermore, investigating the roles of other intratumoral microbiota, such as fungi, in tumorigenesis and immune regulation is of significant importance. Although evidence on the contribution of intratumoral fungi in cancer remains limited, emerging findings suggest that the composition and distribution of intratumoral fungi mirror those of bacteria, varying with tumor location [189]. Notably, certain cancer-specific fungal species, including *Malassezia* [190], *Alternaria alternata* [191], *Aspergillus sydowii* [192], and *Candida albicans* [193], have been identified to accelerate tumor development. An additional critical perspective is the existence of fungus-bacteria-immune interactions in cancer. Lian Narunsky-Haziza and colleagues identified fungal-bacterial-immune clusters driven by fungal co-occurrences, which elicit distinct host immune responses. These clusters are associated with heightened inflammation, lymphocyte exhaustion, and pronounced macrophage activity, and they can predict improved overall survival (OS) [189]. Similarly, in gastrointestinal malignancies, *Candida* and *Saccharomyces* serve as predictors of host gene expression patterns and exhibit extensive interactions with *Lactobacillus* species. Additionally, tumors enriched with *Candida* display a pro-inflammatory immune profile, characterized by upregulated genes involved in cytokine signaling, immune responses, and inflammation. Furthermore, patients with elevated *Candida* infection rates exhibit significantly reduced survival, indicating that *Candida* could serve as a promising prognostic biomarker for gastrointestinal cancer [194]. The emergence of cancer-specific fungi provides a promising starting point for investigating how intratumoral fungal symbiosis contributes to tumorigenesis through complex mechanisms, potentially paving the way for innovative therapeutic strategies and improved prognostic outcomes for cancer patients.

Some questions regarding the intratumoral microbiomes also need to be resolved. First, it remains unclear whether intratumoral microbiomes adjust their genomes or affect the host genomes to adapt to the tumor microenvironment. Further research should explore how they survive inside cells in the long term. Second, it is unknown whether different immunomodulatory pathways are involved in the regulation of unique intratumoral microbiomes in different tumor types. Despite the consensus that certain intratumoral microbiota play a causal role in tumor development, many questions remain unanswered. For example, how do specific microbes and microbial communities promote or inhibit tumor occurrence? In the future, more efforts should be made to elucidate the mechanisms underlying the role of intratumoral microbiomes in tumor development.

## Conclusion

In conclusion, the tumor microbiome is a critical component of the tumor microenvironment, significantly influencing cancer progression and immune responses. This review highlights three key points: first, advanced methodologies now allow for a more comprehensive exploration of the tumor microbiome, especially newly developed sequencing-based technologies, which provide innovative research methods for in-depth analysis of intratumoral microbiome. Second, specific microbial species have been identified as either malignancy promoters (*F. nucleatum, B. fragilis, P. gingivalis*) or protective agents (*A. muciniphila, Lactobacillus, Clostridium*), underscoring their dual role in tumor immune regulation. Lastly, manipulating the tumor microbiome presents a promising strategy for enhancing the effectiveness of cancer therapies. Future research should focus on integrating microbiome-targeted interventions into broader cancer treatment frameworks to improve therapeutic outcomes.

## Data Availability

Not applicable.

## Abbreviations

CEACAM1: Carcinoembryonic antigen-related cell adhesion molecule 1
CAST-R-HP: Clinical Antimicrobial Susceptibility Test Ramanometry for *H. pylori*
CSF1-3: Colony-stimulating factor 1–3
*F. nucleatum*: *Fusobacterium nucleatum*
CRC: Colorectal cancer
c-FIB/SEM: Correlative focused ion beam/scanning electron microscopy
CLEM: Correlative light and electron microscopy
CXCL1: C-X-C motif chemokine ligand 1
*B. fragilis*: *Bacteroides fragilis*
ETBF: Enterotoxigenic *B. fragilis*
CIMP: CpG island methylator phenotype
MLH1: MutL homolog 1
*H. pylori*: *Helicobacter pylori*
VacA: Vacuolating cytotoxin A
CagA: Cytotoxic- associated gene A
OipA: Outer inflammatory protein A
*P. gingivalis*: *Porphyromonas gingivalis*
CXCR4: C-X-C chemokine receptor type 4
*A. muciniphila*: *Akkermansia muciniphila*
I3A: Indole-3-acetic acid
IPA: Indole-3-propionic acid
*C. butyricum*: *Clostridium butyricum*
SCFAs: Short-chain fatty acids
GSH: Glutathione
DFS: Disease-free survival
FMT: Fecal microbiota transplantation
FISH: Fluorescence in situ hybridization
ICI: Immune checkpoint inhibitor
IF: Immunofluorescence
IHC: Immunohistochemistry
ILFs: Isolated lymphoid follicles
INVADEseq: Invasion-adhesion-directed expression sequencing
LPS: Lipopolysaccharides
LTA: Lipoteichoic acid
MiPACT-HCR: Microbial identification using the passive clarity technique with hybridization chain reaction
MicroSPLiT: Microbial split-pool ligation transcriptomics
MDSCs: Myeloid-derived suppressor cells
NK cell: Natural killer cell
NACI: Neoadjuvant chemoimmunotherapy
NOD1: Nucleotide-binding oligomerization domain containing 1
NLRP3: NOD-like receptor family pyrin domain containing 3
OMVs: Outer membrane vesicles
OS: Overall survival
PD-L1: Programmed cell death ligand 1
PD-1: Programmed cell death protein 1
PEHPSI: Prokaryotic and eukaryotic cell hybrid probes for in situ imaging
PETRI-seq: Prokaryotic Expression-profiling by Tagging RNA In Situ and sequencing
SEM: Scanning electron microscopy
TIGIT: T cell immune receptors with immunoglobulin and ITIIM domain
TiDaL: Tissue clearance and d-amino acid labeling
TEM: Transmission electron microscopy
TME: Tumor microenvironment
TAMs: Tumor-associated macrophages
